# Surface-site reactivity in small-molecule adsorption: A theoretical study of thiol binding on multi-coordinated gold clusters

**DOI:** 10.3762/bjnano.7.6

**Published:** 2016-01-18

**Authors:** Elvis C M Ting, Tatiana Popa, Irina Paci

**Affiliations:** 1 Department of Chemistry, University of Victoria, Victoria, BC, V8W 3V6, Canada

**Keywords:** coordination, gold clusters, methylthiol, sulfur–gold interactions, thiol adsorption

## Abstract

**Background:** The adsorption of organic molecules on metal surfaces has a broad array of applications, from device engineering to medical diagnosis. The most extensively investigated class of metal–molecule complexes is the adsorption of thiols on gold.

**Results:** In the present manuscript, we investigate the dependence of methylthiol adsorption structures and energies on the degree of unsaturation at the metal binding site. We designed an Au_20_ cluster with a broad range of metal site coordination numbers, from 3 to 9, and examined the binding conditions of methylthiol at the various sites.

**Conclusion:** We found that despite the small molecular size, the dispersive interactions of the backbone are a determining factor in the molecular affinity for various sites. Kink sites were preferred binding locations due to the availability of multiple surface atoms for dispersive interactions with the methyl groups, whereas tip sites experienced low affinity, despite having low coordination numbers.

## Introduction

The interactions between organic molecules and metallic surfaces have been the subject of significant interest in recent years, because of their fundamental relevance in a broad array of nanoscience applications. One area of interfacial research has focused on the binding properties of aminoacids and peptides on metal substrates [1–12], due to the relevance of these interactions in device fabrication for biological sensors [13–16], for in vivo nanoparticle imaging, tagging and tracing [17–20], and for developing a general understanding of nanoparticle biosafety [21–22], among other issues. The sulfur-containing aminoacids cysteine, homocysteine and methionine are often targeted for such investigations, because of the binding affinity of sulfur-based groups to metal atoms.

Much is known of the adsorption behavior of sulfur-containing aminoacids on low-Miller-index [(111), (100), (110)] gold and silver surfaces [3,23–35], though many questions regarding binding modes and condensed phase behavior still remain. Quantum calculations have been used to examine the behavior of single molecules or small molecular clusters on ideal surfaces [23–27,33–34,36–37]. Several classical simulation studies also exist, generally focused investigating peptide behavior at a metal surface [38–40]. A still outstanding issue is the change in aminoacid or peptide behavior upon adsorption on non-ideal surfaces, i.e., substrates with adatoms, high-Miller-index facets or surface curvature have been examined individually [41–45], but no rigorous studies of the dependence of surface binding on site reactivity or coordination have so far been done for aminoacids.

That unsaturated metal atoms bind more strongly to adsorbates has been long established in the literature, particularly as a result of nanoparticle–molecule studies. Several thorough studies by Nørskov and co-workers [46–48] investigated site coordination effects on the catalytic oxidation on Pt and Au nanoclusters, seeking to elucidate the experimentally observed dependence of catalytic activity on nanoparticle size and shape. A recent experimental study by Mostafa et al. [49] convincingly argued that the catalytic activity of Pt nanoparticles for the oxidation of 2-propanol was positively correlated to not only the average unsaturation of the surface metal atoms, but also to the fraction of edge and corner atoms, which present the lowest coordination.

With an end goal of developing a coordination-dependent description of aminoacid-surface interactions, we focus, in the current work, on the surface-binding groups. To this end, we replaced molecular backbones with methyl groups, in order to remove factors such as lone-pair or charge-group surface interactions, and the overall backbone–surface interaction. We designed a 20-atom Au cluster, with gold atom sites spanning an array of coordination numbers N*_i_*, with *i* = 3–9, and investigated the binding behavior of single molecules of methylthiol in its non-dissociated and dissociated forms, as a model for the surface-binding group of physisorbed and chemisorbed thiols. The cluster itself was built to model various coordination sites that may be found on planar substrates, or along the surface of much larger Au clusters, and was thus held unoptimized, as discussed in some detail below.

The adsorption of methylthiol, and other thiols, at Au substrates has been the focus of a large number of studies, given its broad relevance in spectroscopy, sensing, nanotechnology and biophysics. Many reviews have been written in the last two decades on the topic of thiol adsorption on gold flat surfaces and clusters [50–62], and on that of aminoacid–gold binding [3,10,63–69]. We refer the reader interested in the broader field to these works, as well as many article collections and dedicated journals, and discuss here primarily those studies directly related to the principal focus of the current work: the dependence of binding structures and stabilities on metal site coordination, in the reduced model of methylthiol–Au_20_ interaction. We find that, despite the limited size of the adsorbate, dispersive interactions play an important role in determining preferred adsorption sites. The strongest adsorption occurred at sites that were relatively unsaturated, but also provided sufficient neighboring surface atoms available to interact dispersively to the molecular backbone.

## Experimental

**Configurational sampling.** Zero-temperature DFT calculations suffer from an inability to broadly sample the configurational space, and are often trapped close to the initial (user input) configuration. This limitation can be particularly problematic in cases where there is an overwhelming contributor to the potential energy surface such as in the case of molecule–surface interactions [70]. An elegant workaround is to use thermal energy as provided by an ab initio molecular dynamics methodology, but this is computationally unfeasible except for small systems. Our group has chosen to provide a broad set of input geometries, often selected via a separate set of classical calculations [23].

**Figure 1:**
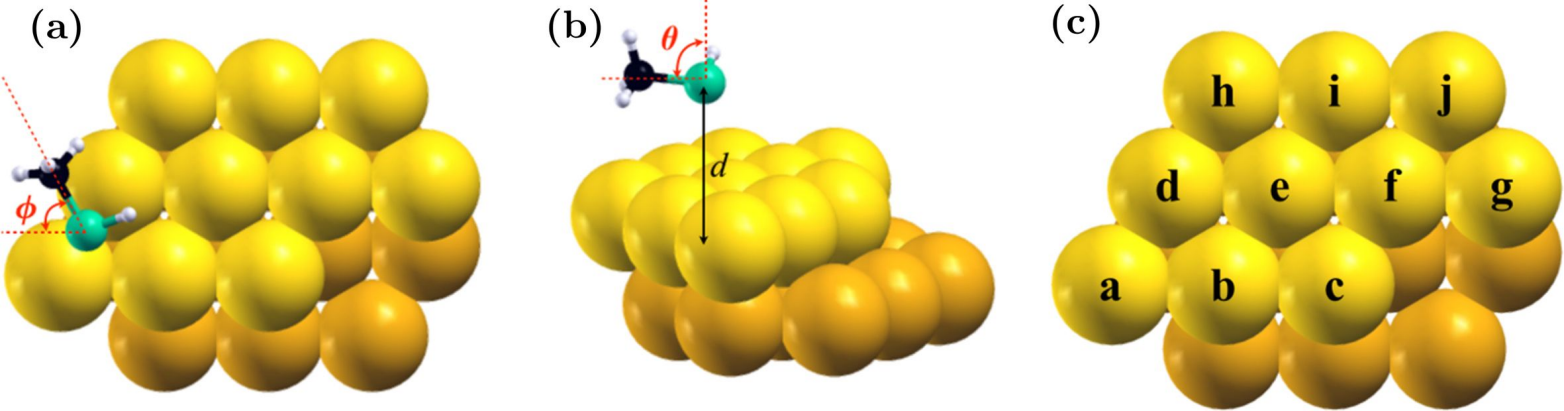
Configurational variables sampled and cluster geometry. In (a) and (b), *d* is the sulfur–gold distance, θ is the polar angle (90° in each initial configuration), and 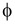 is the azimuthal angle. In (c), the site nomenclature is given in letters.

Here, we perform a systematic scan of possible initial structures by sampling the configurational space through four variables: the distance from the adsorbate headgroup to a gold binding site *d*, the polar angle θ (the angle between the principal axis of the adsorbate and the axis normal to the binding surface), the azimuthal angle 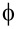 (describing the in-plane orientation of the projection of the adsorbate principal axis), and the relative location of the headgroup with respect to the substrate atoms (Figure 1). Three initial molecule–surface distances were used for all adsorbates (4.1, 4.3 and 4.5 Å), equilibrating to an average S–Au distance of 2.5 Å. Initial configurations were parallel to the surface plane (θ = 90°), and the in-plane angle 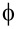 was sampled in 12 increments of 30°. Initial headgroup locations were considered at both bridge and atop sites. On the Au_20_ cluster, ten atop sites and 18 bridge sites were considered for each molecule. Overall, 672 initial structures were considered for each of methylthiol and methylthiolate. Additional calculations were performed for low-coordinated binding sites, to ensure proper sampling of the configurational space. The most stable equilibrated configurations, their binding energies and bond lengths for the different binding sites are discussed in the following pages.

The small Au cluster was designed as a cut from an ideal Au fcc lattice, built to exhibit a variety of kink, edge and surface binding sites, with a broad array of coordination numbers. In order to preserve the designed coordination numbers, the cluster was held unoptimized in the calculations presented here. This approximation neglects the significant relaxation effect of the adsorbate on substrate geometry, but was necessary in order to avoid reorganization of the cluster to a more spherical shape. Such reorganization would preclude the study of the site coordination dependence of adsorption energies and structures, which is the subject of the present work. Below, the coordination number (CN) refers to the numbers of gold-atom neighbors around a particular gold atom. For example, a CN(9) binding site (a central atom on a (111) surface) has nine gold atoms around the central gold atom. On the Au_20_ cluster, only the 10 gold binding sites on the top layer were studied (labeled a–j in Figure 1c).

**Density functional theory (DFT) methodology.** Calculations were performed using the generalized gradient approximation-based Perdew–Burke–Ernzerhof (PBE) functional [71] with and without dispersion corrections, in the SIESTA 3.2 package [72–73]. Core electrons were described in terms of the core pseudopotentials constructed in the scheme of Troullier and Martins [74] with relativistic corrections for the gold atoms. Valence electrons were described using pseudo-atomic orbitals with a polarized triple-ζ (TZP) basis set. The pseudo-atomic orbitals (PAOs) in SIESTA are strictly localized and fall to zero outside a given cut-off radius, chosen by specifying a value of energy shift. Previous calculations by our group and others [36,75] suggested 1 mRy energy shift (equivalent to 6 Å for the cutoff radius of the carbon PAO with the smallest ζ).

A Grimme-type medium range van der Waals correction was included in the PBE-D2 calculations [76]. Dispersion coefficients and van der Waals radii for H, C, N, and S were taken from the original Grimme paper [76], and those for Au, from Liu et al. [77]. The addition of the dispersion correction enhanced non-dissociative binding energies in MeSH by 0.2 to 0.7 eV, and moderately increased chemisorption energies (MeS) by 0.2 to 0.5 eV.

**Non-dissociative adsorption.** The binding energy in non-dissociative adsorption was calculated as the enthalpy of the binding process:

[1]
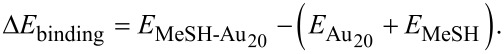


For unbound systems, the basis set superposition error (BSSE) was estimated using a counterpoise (CP) correction:

[2]
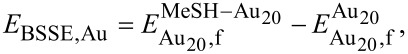


[3]
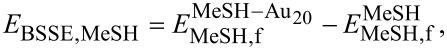


where f indicates the final geometry, subscripts indicate the molecule being considered, and the superscripts indicate the basis set in which each energy was evaluated. *E*_BSSE_ is a negative value. Applying the CP correction to the binding energy, one gets

[4]
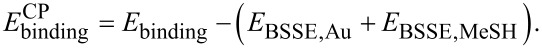


**Dissociative adsorption.** Calculated binding energies for the dissociative adsorption took into account the release of hydrogen as H_2_:

[5]
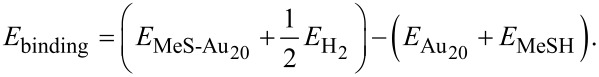


BSSE corrections were not calculated for the dissociative adsorption case, as considering the complex in the molecular (gold + adsorbate) basis set is appropriate in the context of chemical bonding of the two.

## Results and Discussion

### Dissociative binding of methyl thiol on the Au_20_ cluster

Despite the small size of the molecular backbones considered in this study, dispersive interactions held an important place in determining binding methylthiolate/Au binding strengths. As illustrated by the binding data in Table 1, the adsorption energy did not follow a direct, monotonous relationship with the degree of unsaturation of the gold atoms at the binding site. Instead, a convolution of site reactivity and van der Waals attraction was found to determine the preference of methylthiolate to one or another binding site. As a result, adsorption was strongest at the kink sites (**cf** and **fg**), at edge sites where strong methyl-Au dispersive interactions could be established with the bottom layer (**bc**), and at sites where strong unsaturation was supplemented by favorable but weaker dispersive interactions (**ij**, **hi** and **dh**). On the other hand, strongly unsaturated tip sites with fewer neighboring gold atoms (**ab**, **ad** and **jg**) adsorbed weakly.

**Table 1:** Binding energies and relevant distances for methylthiolate–gold adsorption.

site^a^	CN (Au)	*E*_b_ (eV)^b^	(*d*_1_,*d*_2_) (Å)^c^	*N*_neigh._^d^	*d*_C−Au;avg_ (Å)^e^	*E*_m_ (eV)^f^

**ab**	3, 7	−0.62	(2.46, 2.49)	4	3.82	−0.30
**ab**	3, 7	−0.60	(2.45, 2.49)	3	3.54	−0.28
**ab**	3, 7	−0.58	(2.44, 2.47)	3	3.61	−0.26
**bc**	7, 6	−1.06	(2.45, 2.43)	4	3.54	−0.40
**cf**	6, 8	−1.16	(2.42, 2.49)	7	3.80	−0.57
**cf**	6, 8	−1.14	(2.40, 2.46)	6	3.71	−0.52
**cf**	6, 8	−1.12	(2.44, 2.47)	5	3.91	−0.43
**fg**	8, 4	−0.92	(2.53, 2.44)	7	3.79	−0.55
**ad**	3, 6	−0.65	(2.44, 2.49)	4	3.74	−0.29
**dh**	6, 4	−0.95	(2.47, 2.41)	4	3.73	−0.30
**hi**	4, 5	−0.95	(2.42, 2.46)	4	4.03	−0.21
**ij**	5, 4	−1.00	(2.47, 2.40)	4	3.72	−0.28
**gj**	4, 4	−0.78	(2.41, 2.48)	3	3.46	−0.28

^a^The indices of the gold atoms are as indicated in Figure 1c. Several equilibrated structures are indicated for some of the binding sites, in decreasing order of their binding energies. ^b^Binding energy calculated by Equation 5. ^c^Distances from the sulfur atom to the two Au binding sites. ^d^Number of neighboring atoms (at less than 5 Å from the methyl group) available for dispersive interactions. This number includes the two Au atoms bound to the sulphur. ^e^Average distance from the carbon atom to the neighboring Au atoms. ^f^The Molecular Mechanics energy as reported by SIESTA, describing overall dispersive interactions.

The strongest binding arose at the relatively well-coordinated kink site, **cf**, with the methyl group aligned in an equatorial position, and interacting with the bottom layer of the cluster. Coordination numbers at site **cf** were relatively high (6 and 8), with 7 neighboring atoms located at less than 5 Å of the methyl group and available for dispersive interactions. In contrast, the low-coordinated tip site **ad** had coordination numbers 3 and 6, with only 4 neighboring atoms (see Figure 1) within 5 Å of the carbon atom, but showing weak dispersive and overall binding energies. The importance of dispersive interactions of the methyl group in establishing binding site preference is illustrated by the strong correlation between the overall binding energy (*E*_b_ in Table 1) and the additional dispersive interaction of the PBE-D2 formalism (*E*_m_ in Table 1) in this case.

**Figure 2:**
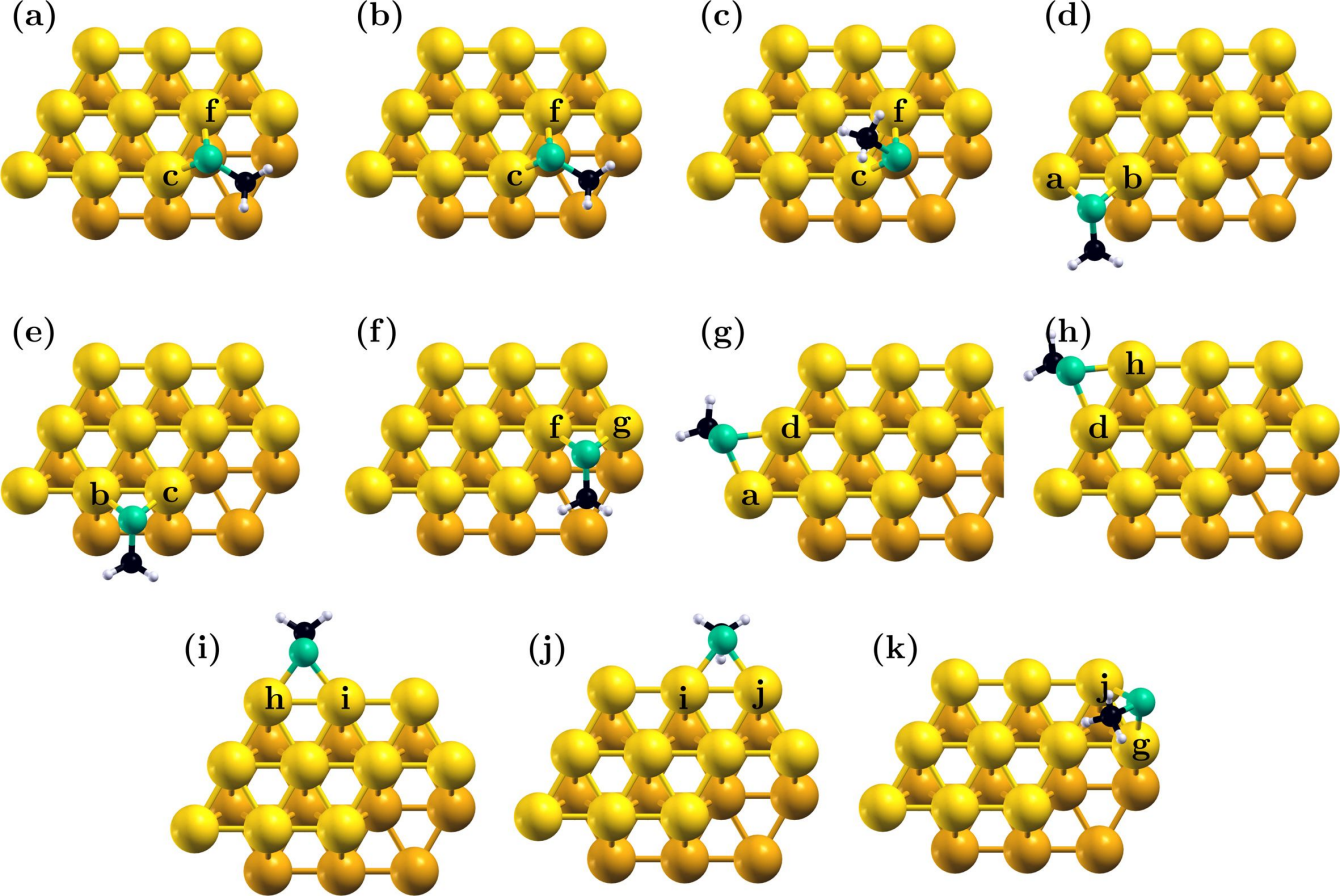
The most stable binding configurations for binding sites on the model Au_20_ cluster. The figures indicate structure for sites **cf** (a–c), **ab** (d), **bc** (e), **fg** (f), **ad** (g), **dh** (h), **hi** (i), **ij** (j) and **gj** (k). For site **cf**, the three most stable configurations are presented in panels a–c, in the order of decreasing stability. Binding site atoms are indicated in the figure.

Regardless of binding site, the thiol group adsorbed to the Au_20_ cluster in a two-bond, off-bridge configuration. In general, Au–S bond lengths were between 2.4 and 2.5 Å (see Table 1), with the sulfur atom generally located roughly along one of the gold lattice planes that intersect the two layers. The relative location of the thiolate group was determined to a great degree by the optimization of the methyl group interactions. The structures of the most stable configurations reported in Table 1 are shown in Figure 2.

Methyl groups experienced weak dispersive binding to Au atoms in the facets neighboring the binding site, with nearest C–Au distances around 3.3–3.7 Å in the most stable configurations. Table 1 includes the number of neighboring Au atoms (atoms within 5 Å of a methyl group), and their average C–Au distance. Several minima were found within thermal energy of the most stable adsorption configuration, with very different methyl group orientations, but similar binding distances. At the strongly binding **cf** kink site, a configuration with the methyl group pointing away from the kink, but interacting with the top layer of surface atoms, experienced only a 0.04 eV energy penalty, whereas slight changes in the location of the methyl group relative to the surface carried a 0.02 eV penalty (see Figure 2a–c). It should be specified that, although multiple low-energy configurations could be found for each site, with the methyl group interacting with various regions of the substrate, the full methyl group desorption (an upright configuration still bound at the thiol end) carried an energetic cost between 0.8 and 1.2 eV. This was consistent with experimental and computational reports of methane adsorption on gold surfaces [78–79].

### The importance of dispersive corrections in chemisorbed systems

Given the importance of dispersive interactions in the adsorption behavior of methylthiolate on Au as emphasized in the previous section, it was deemed necessary to incorporate dispersive corrections in the DFT formalism we employed. However, uncorrected DFT also includes some dispersion. To understand the actual impact of the correction on the observed adsorption behavior, a series of binding energies obtained using PBE without the van der Waals correction were also calculated and are provided in Table 2. As illustrated in the table, binding energies obtained using the pure PBE functional were weaker and much more homogeneous across the various binding sites.

**Table 2:** Methylthiolate–gold binding energies estimated without dispersive corrections.

site^a^	*E*_b_ (eV)

**ab**	−0.33
**bc**	−0.65
**cf**	−0.71
**fg**	−0.45
**ad**	−0.42
**dh**	−0.69
**hi**	−0.74
**ij**	−0.75
**gj**	−0.56

^a^Headings are identical to those described in Table 1.

Whereas S–Au binding distances were relatively unchanged from the PBE-D2 methodology discussed above, the methyl group experienced significant location changes in the PBE approach: A case in point is made by the comparison of the PBE ground state of the **cf**-bound methylthiolate, which had the methyl group located above the surface. This structure was somewhat similar to the third-lowest binding configuration of the PBE-D2-calculated complex (also with the methyl group above the cluster top facet, see Figure 2c and Table 1). In the uncorrected PBE **cf** structure, 5 nearest neighbors could be found within 0.5 Å of the carbon atom, but the structure was more upright, with a larger average C–Au distance (by 0.15 Å). Despite the empirical character of DFT-D2 methods, these results indicate that inclusion of dispersion terms is essential in order to obtain reasonable binding strengths and molecular orientations, for even the simplest molecule–surface complexes.

### Non-dissociative adsorption: the methylthiol form

**Figure 3:**
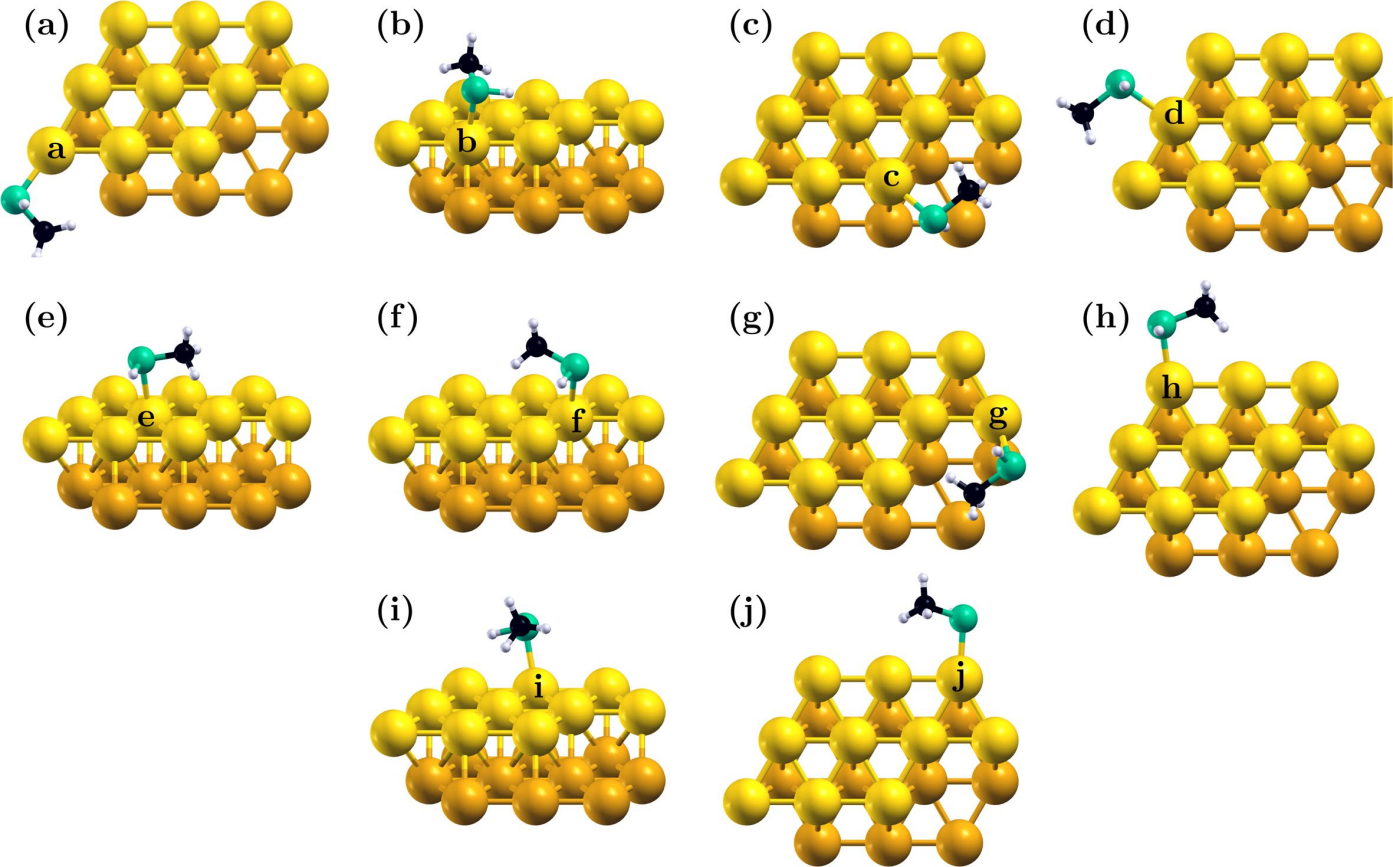
The most stable binding configurations for binding sites on the model Au_20_ cluster. The figures indicate structure for sites **a**–**j** (panels (a–j), respectively). Binding site atoms are indicated in the figure.

Depending on adsorption conditions, and particularly on less reactive surfaces, many thiols maintain their mercapto group undissociated [80–81]. The thiol group then binds to the gold substrate through a single surface atom [23]. We examined here the behavior of complexes of undissociated methylthiol and our Au_20_ cluster. The single-bond configuration provided significantly more orientational freedom to the adsorbate, as illustrated in Figure 3. This in turn allowed both the methyl group and the mercapto hydrogen to approach the surface closely, resulting in overall stronger dispersive interactions relative to the thiolate case (See *E*_m_ in Table 1 and Table 3).

**Table 3:** Binding energies and relevant distances for methylthiol–gold physical adsorption.

site^a^	CN (Au)	*E*_b_ (eV)^b^	*d* (Å)	*N*_neigh._	*d*_C−Au;avg_	*E*_m_ (eV)

**a**	3	−1.04	2.44	4	4.16	−0.23
**b**	7	−1.08	2.51	6	3.96	−0.57
**c**	6	−1.43	2.48	7	3.90	−0.68
**d**	6	−0.70	2.56	3	3.95	−0.31
**e**	9	−0.74	2.67	5	3.88	−0.63
**f**	8	−0.92	2.55	6	4.06	−0.57
**g**	4	−1.26	2.46	6	3.69	−0.54
**h**	4	−0.94	2.47	2	3.45	−0.26
**i**	5	−0.75	2.64	5	3.91	−0.47
**j**	4	−1.03	2.45	2	3.44	−0.27

^a^If not otherwise specified, headings are they same as in Table 1. ^b^Binding energies were calculated by Equation 4.

The case of thiol (non-dissociative) adsorption is a clear example of the duality of thiol group–methyl group binding in these systems. The weaker thiol–gold bond, with single coordination, was more free to move in response to favorable methyl binding conditions. This was apparent in a disconnect between the dispersive added energy (*E*_m_) and the binding energy trends, which had been well correlated in the case of thiolate adsorption. As shown in Table 3, systems where dispersive forces were relatively high could be overall more weakly bound when compared to systems where dispersive interactions are relatively weak: for example, binding at atoms **h** and **i**, in Table 3, where the dispersive and binding energies were negatively correlated. On the other hand though, strong dispersive forces in the **i**-bound thiol were due primarily to methyl–surface interactions, as the relatively long bond length indicated that the thiol group had moved away from the surface. Similar observations can be made for binding at sites **d**, **e** and **f**, with strong overall dispersive forces but relatively long binding distances. The strongest bonds were formed when both of the groups could be satisfied (at sites **c** and **g**, for example.)

**Figure 4:**
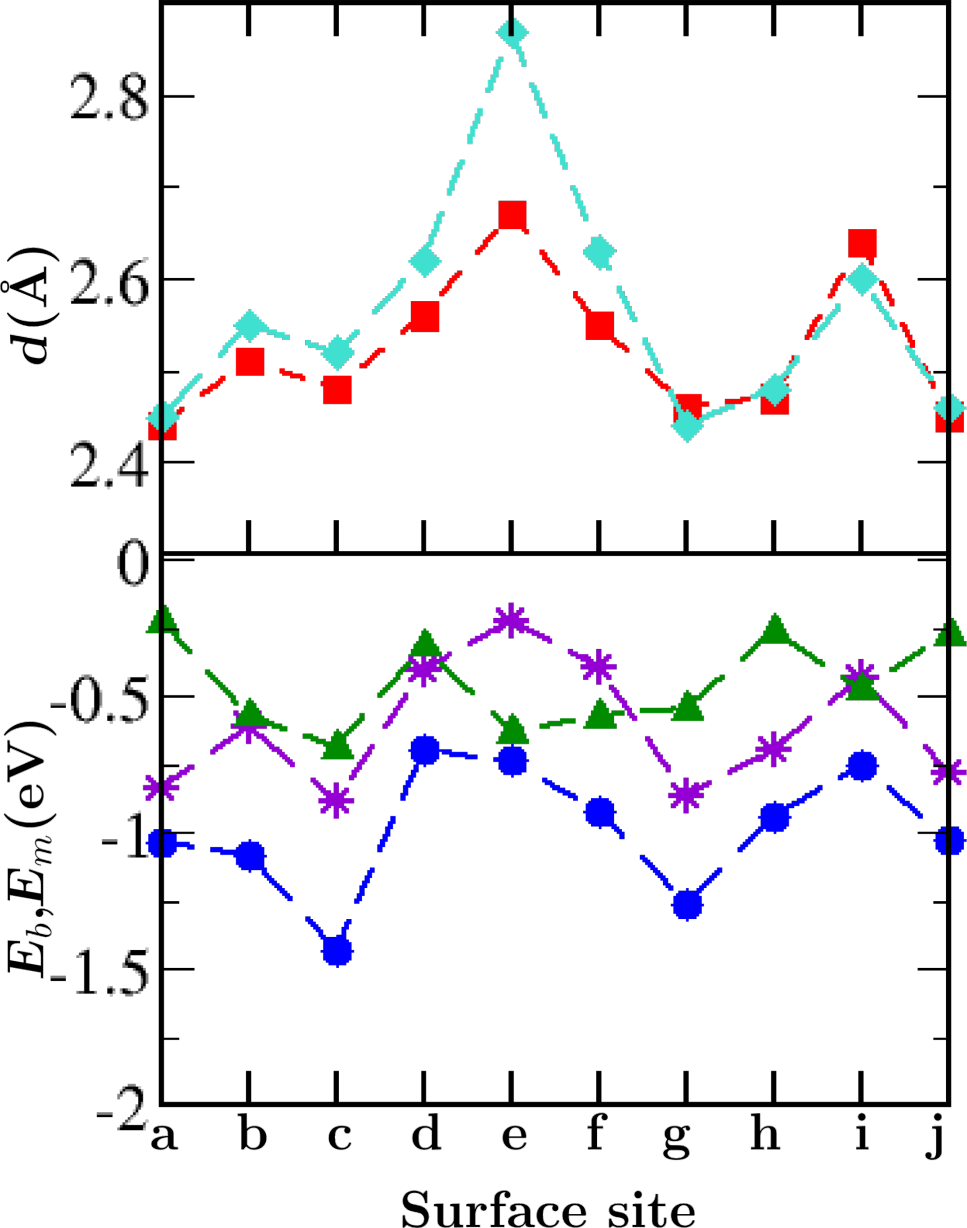
The importance of dispersive corrections in non-dissociative adsorption. Site symbols are given on the abscissa. D2 corrected and uncorrected S–Au bond lengths are shown by red squares and turquoise diamonds, respectively, in the top graph. vdW-Corrected bond energies, uncorrected bond energies and the D2 contribution to the corrected energy are shown by blue circles, purple stars and green triangles, respectively, in the bottom graph.

In thiol adsorption, the use of dispersion corrections becomes important for describing both the thiol group and the methyl group interaction with the substrate. As shown in Figure 4, uncorrected PBE provides good estimates of bonding strengths and bond lengths only in those systems characterized by relatively strong S–Au bonds (and therefore short S–Au bond distances). The length of weaker bonds was overestimated by uncorrected PBE, as well as the strength of the surface interactions of the methyl group, leading to significant structural changes between the two methodologies.

## Conclusion

In order to develop an understanding of thiol binding on complex-structured metal surfaces, we pursued an investigation of dissociative and non-dissociative adsorption at a model 20-atom Au cluster. The cluster model presented a series of distinct available binding sites, with coordination numbers between 3 and 9. To limit the set of competing interactions involved in the adsorption process, we examined methylthiol–Au complexes in a broad examination of their configurational space. We found that, even for small molecules such as methylthiol, dispersive interactions provide an important component of binding affinity. The molecules bound preferentially to kink sites that provided a maximum number of neighboring substrate atoms available for methyl group stabilization. Despite known issues with the empirical nature of dispersive corrections, their inclusion in calculations was essential in order to access reasonable binding structures, particularly for more weakly-bound molecular groups. In effect, a direct parallel between binding preference and the coordination of the adsorption site cannot be drawn without first examining the binding environment and backbone interactions, for even the simple molecules considered here.
